# Mechanisms of the Regulation and Dysregulation of Glucagon Secretion

**DOI:** 10.1155/2020/3089139

**Published:** 2020-07-21

**Authors:** Arnold N. Onyango

**Affiliations:** School of Food and Nutrition Sciences, Jomo Kenyatta University of Agriculture and Technology, P. O. Box 62000, 00200 Nairobi, Kenya

## Abstract

Glucagon, a hormone secreted by pancreatic alpha cells, contributes to the maintenance of normal blood glucose concentration by inducing hepatic glucose production in response to declining blood glucose. However, glucagon hypersecretion contributes to the pathogenesis of type 2 diabetes. Moreover, diabetes is associated with relative glucagon undersecretion at low blood glucose and oversecretion at normal and high blood glucose. The mechanisms of such alpha cell dysfunctions are not well understood. This article reviews the genesis of alpha cell dysfunctions during the pathogenesis of type 2 diabetes and after the onset of type 1 and type 2 diabetes. It unravels a signaling pathway that contributes to glucose- or hydrogen peroxide-induced glucagon secretion, whose overstimulation contributes to glucagon dysregulation, partly through oxidative stress and reduced ATP synthesis. The signaling pathway involves phosphatidylinositol-3-kinase, protein kinase B, protein kinase C delta, non-receptor tyrosine kinase Src, and phospholipase C gamma-1. This knowledge will be useful in the design of new antidiabetic agents or regimens.

## 1. Introduction

The hormone glucagon, produced by pancreatic alpha cells, contributes to the regulation of blood glucose by promoting hepatic glucose production in response to declining blood glucose. However, its excessive secretion contributes to the development of type 2 diabetes [1, 2]. Moreover, in both type 1 and type 2 diabetes, its secretion is dysregulated; with hypersecretion at moderate and high glucose, aggravating hyperglycemia; and failure of secretion at low glucose, leading to life-threatening hypoglycemia [2, 3]. The mechanisms of such alpha cell dysregulations are not well understood. This article discusses the related literature to present an up-to-date understanding of these processes, beginning with an outline of the pathways of glucagon secretion. The mechanism of induction of hyperglucagonemia in otherwise healthy individuals and how this contributes to type 2 diabetes is discussed. A synthesis of the literature unveils a signaling pathway that contributes to glucose- and/or hydrogen peroxide-induced glucagon secretion. Excessive activation of this pathway in diabetes dysregulates glucagon secretion through alpha cell oxidative stress and reduced ATP synthesis. The relevance of such a pathway to the antihyperglycemic and antihypoglycemic effects of some antidiabetic agents is discussed.

## 2. Glucagon Secretion Pathways

Glucagon synthesis involves transcription of the preproglucagon (*Gcg*) gene to produce proglucagon mRNA, which is translated to proglucagon, whose cleavage by prohormone convertase 2 produces glucagon [4]. The synthesized glucagon molecules are packaged into secretory vesicles (SVs), which need to be translocated to the plasma membrane (PM) where they get docked through protein-protein interactions [5, 6]. Before secretion, the vesicles get primed for exocytosis through protein interactions that promote their rapid calcium-dependent fusion with the PM [5].

**Figure 1:**
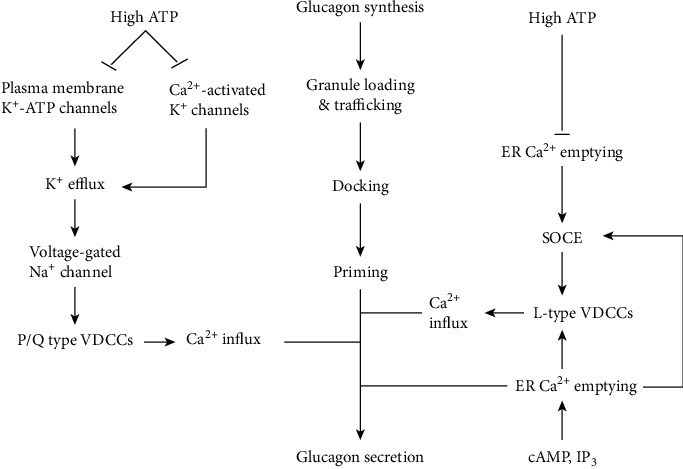
Pathways of glucagon secretion. VDCC: voltage-dependent calcium channel; ER: endoplasmic reticulum; SOCE: store-operated calcium entry.

As illustrated in Figure 1, one of the hypothesized pathways leading to primed glucagon granule exocytosis begins with potassium efflux through ATP-dependent K^+^ channels (K-ATP channels) [7, 8]. According to this hypothesis, glucagon secretion requires the closure of most of these channels, allowing limited K^+^ efflux to alter the membrane potential to a range that permits the opening of voltage-dependent Na^+^ channels; the resulting Na^+^ influx causes subsequent opening of P/Q type voltage-gated calcium channels (VDCCs); calcium influx through these VDCCs is coupled to fusion of glucagon vesicles with the plasma membrane, resulting in glucagon secretion; and too low or too high ATP levels induce excessive opening or closure of the K-ATP channels, respectively, leading to the inhibition of this pathway [7–9]. K^+^ channels activated by intracellular calcium (calcium activated K^+^ channels) were recently found to contribute to glucagon secretion and were suggested to be useful in limiting voltage-dependent inhibition of P/Q type VDCCs during prolonged periods of low glucose [10]. Nevertheless, this K-ATP channel hypothesis is not fully accepted [7, 11]. For example, at low glucose, reduction in alpha cell ATP by inhibition of fatty acid oxidation was found not to affect K-ATP channel conductance, and no membrane hyperpolarization due to K^+^ efflux was observed; instead, there was membrane depolarization due to Na^+^/K^+^ ATPase inhibition [11]. Likewise, as reviewed by Gylfe [7], it has been reported in some studies that glucose, which increases ATP, promotes membrane hyperpolarization rather than the expected depolarization due to K-ATP channel closure.

Other glucagon secretion pathways depend on the reduction of ER calcium content (Figure 1). For example, at low glucose, the ER calcium pump SERCA is relatively inhibited, and the low ER calcium levels cause the ER transmembrane protein, stromal-interacting molecule 1 (STIM 1), to oligomerize and move to interact with the plasma membrane calcium channel orai1, thus activating this ‘store-operated channel' and inducing store operated calcium entry (SOCE) [7, 12]. SOCE depolarizes the membrane, thus inducing calcium entry through L-type VDCCs which promote exocytosis [7, 12]. High glucose inhibits SOCE by reverse translocation of STIM 1 to the ER, and this effect is maximal by 3 mM glucose [7, 12].

ER calcium release and glucagon secretion can be induced even at high glucose subsequent to the intracellular increase in cyclic adenosine monophosphate (cAMP) or inositol triphosphate (IP_3_) [6, 13, 14]. For example, fatty acids promote glucagon secretion at both low and high glucose by binding to the FFAR1 receptor, which is coupled to phospholipase C activation and generation of IP_3_, which induces the release of calcium from the ER, thus raising the cytosolic calcium, which is amplified by SOCE and L-type VDCCs to promote glucagon exocytosis [11, 14–16]. Besides, fatty acids are also metabolized to produce ATP, which is required for a variety of processes, including glucagon synthesis, glucagon vesicle trafficking, docking and priming, maintaining Na^+^/K-ATPase activity for membrane repolarization, and preventing excessive opening of K-ATP channels [8, 11, 14, 17].

## 3. Isolated Alpha Cells Exhibit a V-Shaped Glucagon Secretion Curve in Response to Increasing Glucose: The Influence of ATP and a Signaling Pathway Leading to Phospholipase C Gamma-1 Activation and ER Calcium Release

Isolated alpha cells have a V-shaped glucagon secretion curve in response to increasing glucose concentrations from 0 mM, with maximal suppression at moderate glucose concentrations of 5-7 mM [6, 18]. At low glucose concentrations, ATP plays a signaling role in glucagon secretion through cAMP elevation, which is important for accelerating the mobilization of glucagon granules to the readily releasable pool [13, 19]. Suppression of glucagon secretion in response to glucose has been attributed to increasing ATP concentrations and resultant closure of K-ATP channels, membrane hyperpolarization through increased Na^+^/K^+^ ATPase activity, inhibition of ER calcium release, and reduction of cAMP concentrations [6–8, 13]. cAMP level reduction may be explained by ATP-induced ER filling and reverse translocation of STIM to the ER, since, at the plasma membrane, STIM 1 activates adenylyl cyclase [12, 13]. The increasing ATP also coincides with decreasing activation of adenosine monophosphate kinase (AMPK), a promoter of glucagon secretion by an unknown mechanism [20, 21].

**Figure 2:**
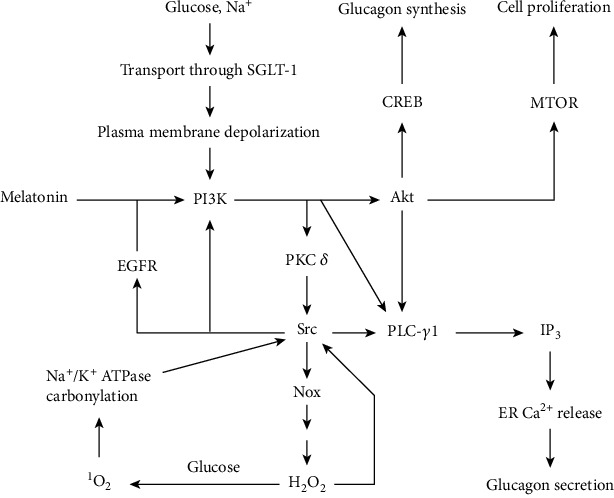
Suggested signaling pathway for glucose-induced increase in glucagon synthesis, glucagon secretion, and alpha cell proliferation. Melatonin and hydrogen peroxide (H_2_O_2_) also initiate the pathway.

The reason for increasing glucagon secretion above the 5-7 mM glucose range is less well understood. However, as suggested hereafter, a signaling pathway beginning with sodium-glucose cotransporter 1 (SGLT-1) and involving the generation of reactive oxygen species (ROS) can explain this phenomenon (Figure 2). This is partly because ROS released from beta cells at 16.7 mM glucose were found to increase alpha cell glucagon content and secretion and alpha cell proliferation [22]. Similarly, hyperglycemia induces alpha cell hydrogen peroxide production, PI3K-Akt signaling, cell proliferation, and glucagon secretion [23]. This is in contrast to the hyperglycemia- and hydrogen peroxide-induced inhibition of PI3K-Akt in beta cells [23]. In the Goto-Kakizaki diabetes-prone rat model, the elevation in pancreatic islet PI3K-Akt is associated with increased activation of the nonreceptor tyrosine kinase Src and related ROS production, which can be inhibited by Src inhibitors [24, 25]. Src activation has similarly been found in pancreatic islets of db/db mice [26]. Inhibitors of the epidermal growth factor receptor (EGFR) were found to reduce ROS in islets from Goto-Kakizaki rats, and it was postulated that Src may transactivate this receptor [25]. In addition, an increase in glucagon secretion in hyperglycemia is associated with an increased activity of protein kinase C delta (PKC-*δ*) [27]. Thus, PI3K-Akt, PKC-*δ*, Src, and EGFR should be important components of the suggested signaling pathway (Figure 2).

According to Figure 2, transport of glucose and sodium (Na^+^) through SGLT-1 is responsible for initiating signaling, through PI3K activation that leads, via PKC-*δ* and Src, to NADPH oxidase (Nox) and the production of hydrogen peroxide (H_2_O_2_). This is based on the analogy that in cardiomyocytes exposed to high glucose, glucose transport through SGLT-1 induces Nox2 activation in a process dependent on sodium and glucose transport but not metabolism, and which is associated with PKC activation [28]. SGLT1 was reported to contribute to glucagon secretion when islets were incubated for 2 hrs with both 5 mM and 20 mM glucose, by a mechanism dependent on transport rather than glucose metabolism [29]. Membrane depolarization, as can be induced by Na^+^ entry through SGLT-1, can trigger activation of PI3K and Akt, upstream of Nox2 [30]. Akt promotes alpha cell proliferation via mammalian target of rapamycin (MTOR) [31] and also activates CREB [32], which promotes glucagon synthesis [4].

Human alpha cells express the melatonin 1 receptor (MT1) [33]. Melatonin signaling through this receptor induces PI3K-Akt signaling [34] and promotes glucagon secretion via PI3K and PLC-*γ*1, even at high glucose such as 16.7 mM [35]. PI3K activates PLC-*γ*1 through production of phosphatidylinositol 3-phosphate, but Akt can also activate this phospholipase, especially when EGFR is also activated [36]. PLC-*γ*1 generates IP3, which causes ER calcium release and glucagon secretion as already described in Glucagon Secretion Pathways. PKC-*δ* promotes trafficking of glucagon secretory granules to sites close to L-type VDCCs that participate in ER-dependent glucagon secretion [37]. Thus, by activating PI3K-Akt, glucose can induce glucagon secretion similarly to melatonin. At increasing glucose above the 7 mM glucose, the ATP level in alpha cells remains constant and maximal [20, 38]. Therefore, the increase in glucagon secretion with increasing glucose may be due to increasing activation of the signaling pathway in Figure 2 rather than changes in ATP. Nox activity may increase with increasing glucose because of higher NADPH availability from the pentose phosphate pathway, since this pathway was found to be required for hyperglycemia-induced Nox activity elevation in cardiomyocytes [28]. Moreover, with increasing glucose, there is increased nonenzymatic protein glycation, which further promotes the activation of Nox and Src [28, 39, 40].

PI3K activates PKC-*δ* [41], which activates Akt, Nox, and Src [41–43]. Src activates Nox, PLC-*γ*1, and EGFR [25, 44, 45]. EGFR activates both PLC-*γ* and PI3-K [36]. Nox produces superoxide anions that are converted by superoxide dismutase to hydrogen peroxide (H_2_O_2_). Hydrogen peroxide, via Src, activates PI3K [46], thus establishing a positive feedback loop for sustained P13-Akt activation and hydrogen peroxide generation. This also explains the fact that hydrogen peroxide can promote glucagon secretion, increase glucagon content, and cell proliferation [22].

**Figure 3:**
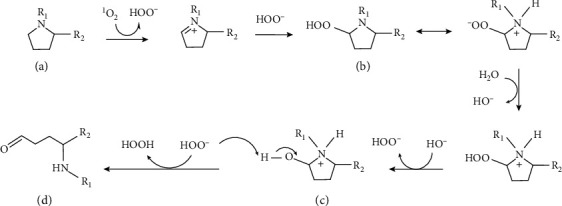
Suggested reaction of proline residue (a) with singlet oxygen (^1^O_2_) to form hydroperoxide (b), which reacts with H_2_O to form hydroxy-derivative (c) and hydroperoxide anion (HOO –), followed by reaction of the latter two to form glutamate 5-semialdehyde residue (d).

Hydrogen peroxide-mediated Src activation depends on sulfenylation of two cysteine residues [47]. ROS-mediated carbonylation of specific proline and threonine residues of Na^+^/K^+^ ATPase additionally promotes Src signaling by freeing the latter from an inhibitory interaction with the former, and this has been reported to be involved in the pathogenesis of obesity and cardiovascular dysfunctions [48, 49]. Although it has been suggested that such carbonylation involves hydroxyl radicals generated by the Fenton reaction between hydrogen peroxide and ferrous ions [48], this is unlikely due to the very high reactivity of hydroxyl radicals, which makes them react unselectively [50]. Singlet oxygen (^1^O_2_) is a more selective ROS, which can be formed by the reaction of hydrogen peroxide with glucose [51], oxidizes amines [52], and such oxidation was recently suggested as being involved in the formation of biologically relevant amide-type adducts such as N*ε*-(hexanoyl) lysine [53]. Thus, it is proposed that the carbonylation of Na^+^/K^+^ ATPase may be mediated by singlet oxygen according to Figure 3.

## 4. Elevated Plasma Nonesterified Fatty Acids (NEFA) Induce Alpha Cell Insulin Resistance and Associated Dysfunctions That Promote the Pathogenesis of Type 2 Diabetes

**Figure 4:**
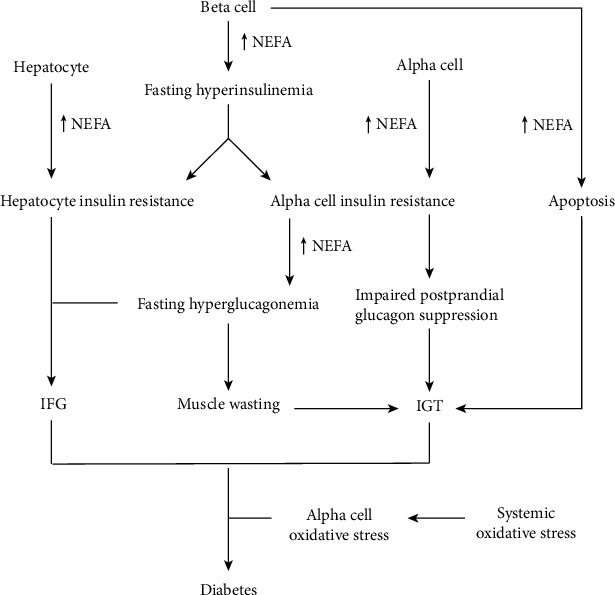
Fatty acid- and oxidative stress-induced alpha cell dysfunction upstream of type 2 diabetes.

Dysregulation of glucagon secretion starts before the development of type 2 diabetes [54, 55]. The path towards type 2 diabetes involves two major types of prediabetic states, namely, impaired fasting glucose (IFG, defined by fasting glucose of 5.6-6.9 mM) and impaired glucose tolerance (IGT, defined by 2-hour glucose of 7.8-11 mM after oral consumption of 75 g equivalent of glucose) [56]. Elevated hepatic glucose production is the key characteristic of IFG and decreased suppression of postprandial hepatic glucose production contributes to IGT [57]. Fatty acids induce a dose-dependent elevation of glucagon secretion at both low- and moderate-glucose concentrations [14, 16, 58]. Thus, conditions such as obesity that elevate plasma NEFA expose alpha cells to the latter's glucagon-elevating effects [59]. However, both glucagon and fatty acids induce insulin secretion [16, 60], which inhibits glucagon secretion. Hence, elevated fatty acids may initially promote fasting hyperinsulinemia but not hyperglucagonemia (Figure 4). Sustained NEFA elevation and resultant hyperinsulinemia can induce insulin resistance in alpha cells, hepatocytes, and other cell types [61–63]. Palmitate induces both insulin resistance and ER stress in alpha cells [61, 64]. ER stress upregulates glycogen synthase kinase 3 (GSK3) [65], which causes insulin resistance by phosphorylating insulin receptor substrate 1 (IRS1), which subsequently undergoes ubiquitination and proteosomal degradation [66].

Insulin inadequately inhibits glucagon secretion in insulin-resistant alpha cells [61], resulting in fasting hyperglucagonemia, which promotes fasting hepatic glucose production and IFG, especially in the setting of hepatic insulin resistance (Figure 4). Fasting hyperglucagonemia may promote muscle wasting and is associated with IGT due to decreased postprandial uptake of glucose by muscles [1, 67]. Likewise, alpha cell insulin resistance reduces postprandial glucagon suppression, and thus sustains hepatic glucose production in the postprandial state, which contributes to IGT [68]. Chronic exposure of beta cells to palmitate induces ER stress and apoptosis [64], which reduces insulin production, thus contributing to IGT. Although alpha cells are subject to ER stress, they are resistant to apoptosis [69]. Thus, the alpha cell to beta cell ratio with associated glucagon to insulin ratio may increase with time, further elevating blood glucose [31].

Prediabetic individuals either revert to normoglycemia or progress to diabetes. Systemic oxidative stress is an important factor associated with progression to diabetes [70–73]. Hypertension is also strongly related to the progression to diabetes [74], and this can be linked to oxidative stress [75]. During systemic oxidative stress, alpha cells may be chronically exposed to hydrogen peroxide from beta cells and endothelial cells, and this may lead to chronic activation of PI3K-Akt signaling in alpha cells according to Figure 2. Although Akt ordinarily phosphorylates and inhibits GSK3, chronic Akt activation desensitizes GSK3 from this inhibition [76]. GSK3 activation promotes mitochondrial damage, including inhibition of complex 1, mitochondrial fission, dissolved cristae, and overall change in morphology [77–80]. Alpha cells with such mitochondrial damage may be under elevated superoxide anion production even at basal glucose [81]. As described in the next section, oxidative stress and mitochondrial alterations increase glucagon secretion at normal and high glucose and are therefore likely to accelerate the occurrence of frank hyperglycemia characteristic of diabetes. Hence, chronic infratherapeutic treatment of Goto-Kakizaki young rats with the GSK3 inhibitor, lithium, prevented islet inflammation and diabetes [82].

## 5. Alpha Cell Insulin Resistance, Mitochondrial Abnormalities, and Chronic Oxidative Stress Dysregulate Glucagon Secretion in Diabetes

In normal pancreatic islets, unlike isolated alpha cells, the rise in glucagon secretion at glucose concentrations above 7 mM is suppressed by paracrine action of somatostatin and insulin produced by beta cells and delta cells, respectively, and by gap junction coupling between these cells [11, 83]; but, this paracrine suppression is lost in diabetes because of alpha cell insulin and somatostatin resistance [18, 55]. Chronic exposure of alpha cells to high glucose upregulates the expression of SGLT-1 [9, 29] and overactivates the signaling pathway in Figure 2, as evidenced by increased activation of PI3K-Akt, PKC-*δ*, Src, and ROS generation in diabetic islets [24–27, 81]. Oxidative stress and mitochondrial abnormalities cause reduced ATP production in alpha cells [9, 84, 85]. Decreased ATP increases glucagon secretion at both moderate and high glucose [9, 85]. On the other hand, at low glucose, ATP can drop below the level required for glucagon secretion, thus leading to failure of glucagon counterregulation and hypoglycemia [9, 84, 85]. Accordingly, the antioxidant epigallocatechin-3-gallate prevented oxidative stress and restored glucagon secretion in a TC1-6 pancreatic alpha cell line, leading to the suggestion that combining conventional antihyperglycemia therapy with antioxidant therapy may avert hypoglycemia in clinical treatment of diabetes [86]. Oral administration of glucose restores glucagon secretion [87]. Apart from its role as a substrate for ATP synthesis, glucose is metabolized through the pentose phosphate pathway to generate NADPH for reduction of oxidized glutathione, and hence alleviation of oxidative stress [88]. Notably, in response to hypoglycemia, activation of the hypothalamic-pituitary-adrenal axis occurs, resulting in release of catecholamines that greatly increase plasma free fatty acids, yet this does not necessarily resolve the hypoglycemia [89]. Although fatty acids may supply energy to prevent hypoglycemia [11], it is likely that they do not efficiently promote hypoglycemia recovery because they cannot resolve the oxidative stress and might even aggravate it.

## 6. Antioxidant Antidiabetic Agents Improve Glucagon Hypersecretion and Hyposecretion

Glucagon-like peptide 1 (GLP-1) and GLP-1 receptor agonists such as exendin reduce hyperglycemia and are associated with lower risk of hypoglycemia [25, 90, 91]. They promote cAMP formation, which, through Epac2, inhibits Src signaling and induces Nrf2 antioxidant response [25, 92]. Thus, by reducing ROS formation, GLP-1 and its receptor agonists increase ATP production [25, 93], to improve glucagon hyposecretion and hypersecretion. G protein-coupled receptor 119 (GPR119) agonists induce glucagon secretion during hypoglycemia but not hyperglycemia in diabetic mice [94]. Like GLP-1R signaling, GPR119 signaling involves cAMP production; and this receptor has the advantage of being robustly expressed in alpha cells, unlike the GLP-1 receptor [94].

Exogenous insulin administration promotes somatostatin secretion, which aggravates hypoglycemia by reducing cAMP formation; while somatostatin receptor antagonists improve hypoglycemia by increased cAMP [3], and associated reduction in oxidative stress. It is likely that, at high glucose, somatostatin only lowers glucagon when in collaboration with insulin, which activates Akt, thus inhibiting GSK3 and promoting Nrf2-associated expression of antioxidant enzymes. Otherwise, somatostatin alone, by reducing cAMP, might induce oxidative stress. Accordingly, diabetes patients, with low insulin secretion, experience hyperglucagonemia although their somatostatin secretion is even upregulated [95].

Although according to Figure 2, SGLT-1 inhibitors should inhibit PI3K-Src-ROS signaling and thus prevent hypoglycemia, this is not the case in [29]. This can be explained by their reduction of glucose entry for ATP synthesis and glutathione reduction. A related result has been reported that in cardiomyocytes, SGLT-1 induces ROS generation at high glucose but promotes survival at low glucose by replenishing ATP stores through enhanced glucose availability [96].

There has been increasing interest in the antidiabetic effects of the flavonoid quercetin, but this has mainly been limited to animal studies [97]. Quercetin has been demonstrated to inhibit glucagon secretion through PKC-*δ* inhibition [27], and it is also known to be an antioxidant and inhibitor of Src [98]. Further studies of this flavonoid and related phytochemicals in the prevention or management of diabetes are warranted. The same applies to the peptide pNaKtide which inhibits Na^+/^K^+^ ATPase-dependent Src activation and has been found to be beneficial against various other metabolic disorders [49].

## 7. Conclusion

In healthy individuals, glucose increases ATP to promote glucagon secretion in hypoglycemia and to suppress glucagon secretion at higher glucose levels to prevent hyperglycemia. At high glucose, such as in the postprandial state, glucagon secretion is suppressed by paracrine action of somatostatin and insulin produced by beta cells and delta cells, respectively. Alpha cell dysfunctions such as insulin resistance, mitochondrial alterations, and oxidative stress contribute to the pathogenesis of type 2 diabetes and glucagon dysregulation in diabetes. A signaling pathway that can be initiated by glucose and sodium transport through SGLT-1 or by hydrogen peroxide promotes glucagon secretion and, if overactivated, may induce oxidative stress and ATP reduction as key contributors to glucagon dysregulation in diabetes. This pathway can be targeted in the search for new antidiabetic agents.
